# Incidence of atrial fibrillation, ischaemic heart disease and heart failure in patients with diabetes

**DOI:** 10.1186/s12933-021-01313-7

**Published:** 2021-06-16

**Authors:** Amy Groenewegen, Victor W. Zwartkruis, Betül Cekic, Rudolf. A. de Boer, Michiel Rienstra, Arno W. Hoes, Frans H. Rutten, Monika Hollander

**Affiliations:** 1 Dept. General Practice, Julius Center for Health Sciences and Primary Care, University Medical Center Utrecht, Utrecht University, Universiteitsweg 100, 3584 CG Utrecht, The Netherlands; 2 Department of Cardiology, University Medical Center Groningen, University of Groningen, Groningen, The Netherlands; 3 University Medical Center Utrecht, Utrecht University, Utrecht, The Netherlands

**Keywords:** Diabetes, Cardiovascular disease, Incidence, Heart failure, Ischaemic heart disease, Atrial fibrillation

## Abstract

**Background:**

Diabetes has strongly been linked to atrial fibrillation, ischaemic heart disease and heart failure. The epidemiology of these cardiovascular diseases is changing, however, due to changes in prevalence of obesity-related conditions and preventive measures. Recent population studies on incidence of atrial fibrillation, ischaemic heart disease and heart failure in patients with diabetes are needed.

**Methods:**

A dynamic longitudinal cohort study was performed using primary care databases of the Julius General Practitioners’ Network. Diabetes status was determined at baseline (1 January 2014 or upon entering the cohort) and participants were followed-up for atrial fibrillation, ischaemic heart disease and heart failure until 1 February 2019. Age and sex-specific incidence and incidence rate ratios were calculated.

**Results:**

Mean follow-up was 4.2 years, 12,168 patients were included in the diabetes group, and 130,143 individuals in the background group. Incidence rate ratios, adjusted for age and sex, were 1.17 (95% confidence interval 1.06–1.30) for atrial fibrillation, 1.66 (1.55–1.83) for ischaemic heart disease, and 2.36 (2.10–2.64) for heart failure. Overall, incidence rate ratios were highest in the younger age categories, converging thereafter.

**Conclusion:**

There is a clear association between diabetes and incidence of the major chronic progressive heart diseases, notably with heart failure with a more than twice increased risk.

**Supplementary Information:**

The online version contains supplementary material available at 10.1186/s12933-021-01313-7.

## Background

Both type 1 and 2 diabetes mellitus (T1DM and T2DM) are important risk factors for the development of cardiovascular diseases (CVD), notably atrial fibrillation, ischaemic heart disease and heart failure [1]. All three are clearly associated with substantial morbidity, reduced longevity [2, 3] and decreased health-related quality of life [4, 5].

The epidemiology of CVD in the general population has been reported to decline. For instance, over the last five decades, the incidence and severity of myocardial infarction (MI) has substantially declined, due to a reduction in (second-hand) smoking and better primary and secondary prevention [6–8]. The incidence of heart failure seems to be stabilizing as well, in part because of the decreased incidence of MI [9]. However, the incidence of atrial fibrillation and heart failure in younger people (< 55 years old) is on the rise [10, 11]. This has, in part, been explained by the dramatic world-wide increase of obesity, [12, 13] although improved awareness and better registration likely also play a role [14, 15].

Obesity and diabetes are strongly linked, and the imminent rise in obesity and diabetes pose a threat in our fights against CV disease. Diabetes, especially T2DM, has strongly been linked to new onset atrial fibrillation, new onset ischaemic heart disease and to new onset heart failure [16–18]. A large cohort study including 1.9 million individuals, recently reported on the occurrence of 12 cardiovascular diseases in patients with and without diabetes, and found that heart failure and peripheral arterial disease are the most common initial manifestations of cardiovascular disease in type 2 diabetes [1].

However, aggregate data on the onset and development of CV disease in diabetic subjects is remarkably scarce. Recent population-based, “real world” estimates are needed, more so now that there are strong indications that newer glucose-lowering drugs have an effect on cardiovascular disease development as well [19]. In this population-based cohort study, we therefore aim to quantify age- and gender-specific incidence of atrial fibrillation, ischaemic heart disease and heart failure, comparing primary care individuals with and without diabetes.

## Methods

### Data source

We performed a dynamic cohort study using routine primary care databases from the Julius General Practitioners’ Network (JGPN). The JGPN is a registration network of general practices in the Netherlands, consisting of nearly 70 general practices and over 370,000 enlisted individuals with 15 to 20 years of follow-up. Patients registered at a participating practice are informed by their GPs and may opt out. The current composition of the database is representative of the Dutch population at large [20] and the database is updated every 3 months.

All Dutch residents are enlisted with a general practitioner (GP), who functions as a gatekeeper for specialist care. Because health care insurance is mandatory and consultation with a GP is covered by the insurance, there is little to no financial threshold for contacting a GP. Furthermore, the majority (~ 84%) of patients with diabetes is enrolled in primary care disease management programs (DMPs) and visits their GPs on a regular basis (two to four times a year) for monitoring of their diabetes and potential cardiovascular complications. General practice registries therefore give a good reflection of cardiovascular disease occurrence in community patients with and without diabetes, at least in the Netherlands. The JGPN database contains structured information on every consultation with an enlisted GP, including information on signs/symptoms, diagnostic testing and patient management. Diagnoses are registered by GPs according to international classification of primary care (ICPC) coding and entered as ‘episodes’, clustering consecutive consultations for the same disease, which is essential for reliable incidence estimates. Diagnoses confirmed in secondary care are reported back to the GP in a digital letter detailing the findings and are entered as ICPC code by the GP. Drug prescriptions are automatically entered in international anatomical therapeutic coding (ATC).

### Study cohort

The study cohort included all individuals aged 40–80 years that were enlisted at one of the participating general practices between January 2014 and February 2019. Because of privacy regulations, only year of birth was available, and day of birth was set on 1 July by default for all participants. The cohort was dynamic, and patients were followed from baseline or from the moment they first enlisted at a participating practice, until they left the cohort due to death, relocation to another region, development of the outcome or until the end of the study.

**Fig. 1:**
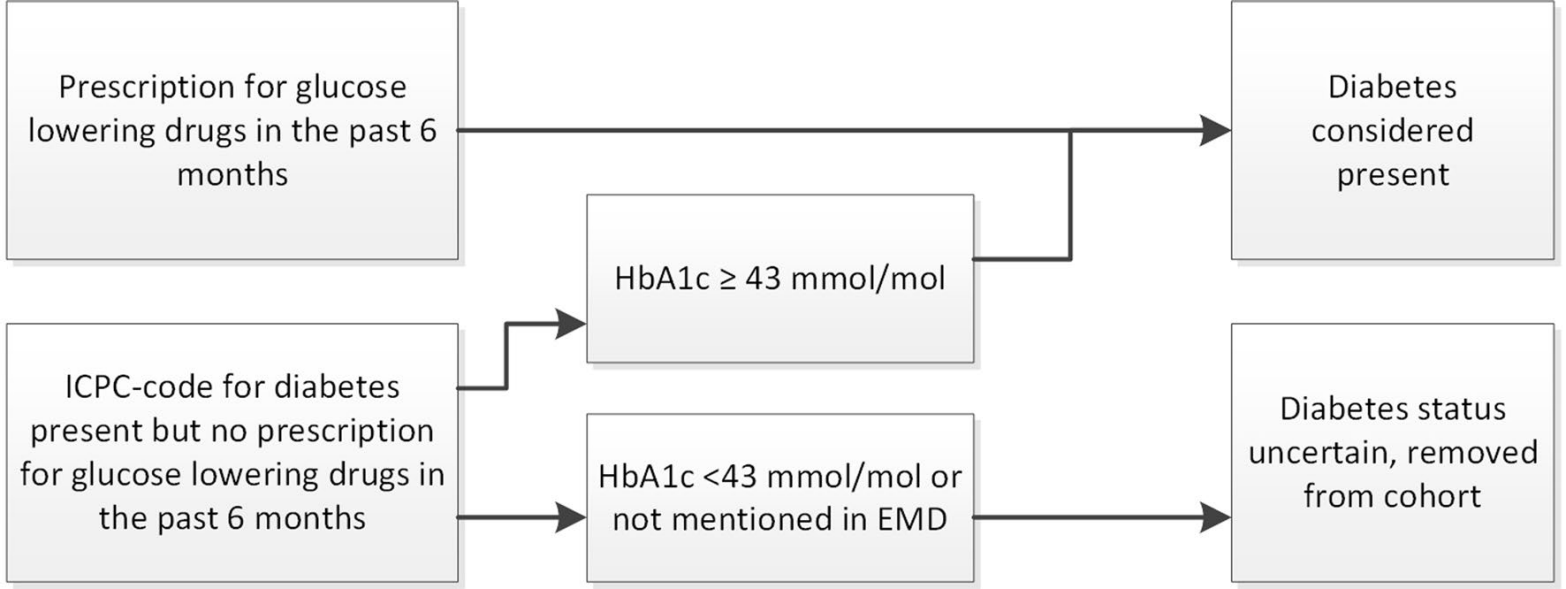
Flowchart of diabetes case validation

The diagnosis of diabetes mellitus was considered to be present in individuals who received a prescription for oral or injectable glucose lowering drugs during 6 months before the inclusion date. HbA1c levels were noted if available. Patients with an ICPC code for diabetes who did not receive any kind of glucose lowering drug and who had completely normal HbA1c levels (< 43 mmol/mol) or in whom HbA1c was never measured, were removed from the cohort as diabetes status was deemed too uncertain. Patients with HbA1c levels ≥ 43 mmol/mol, however, were considered to have diabetes mellitus (see Fig. 1). Because the induction time for cardiovascular diseases due to diabetes is uncertain, patients who developed diabetes during follow-up contributed time at risk in the non-diabetes group until the diabetes index date and were censored at the time of diagnosis.

### Study outcomes

**Fig. 2:**
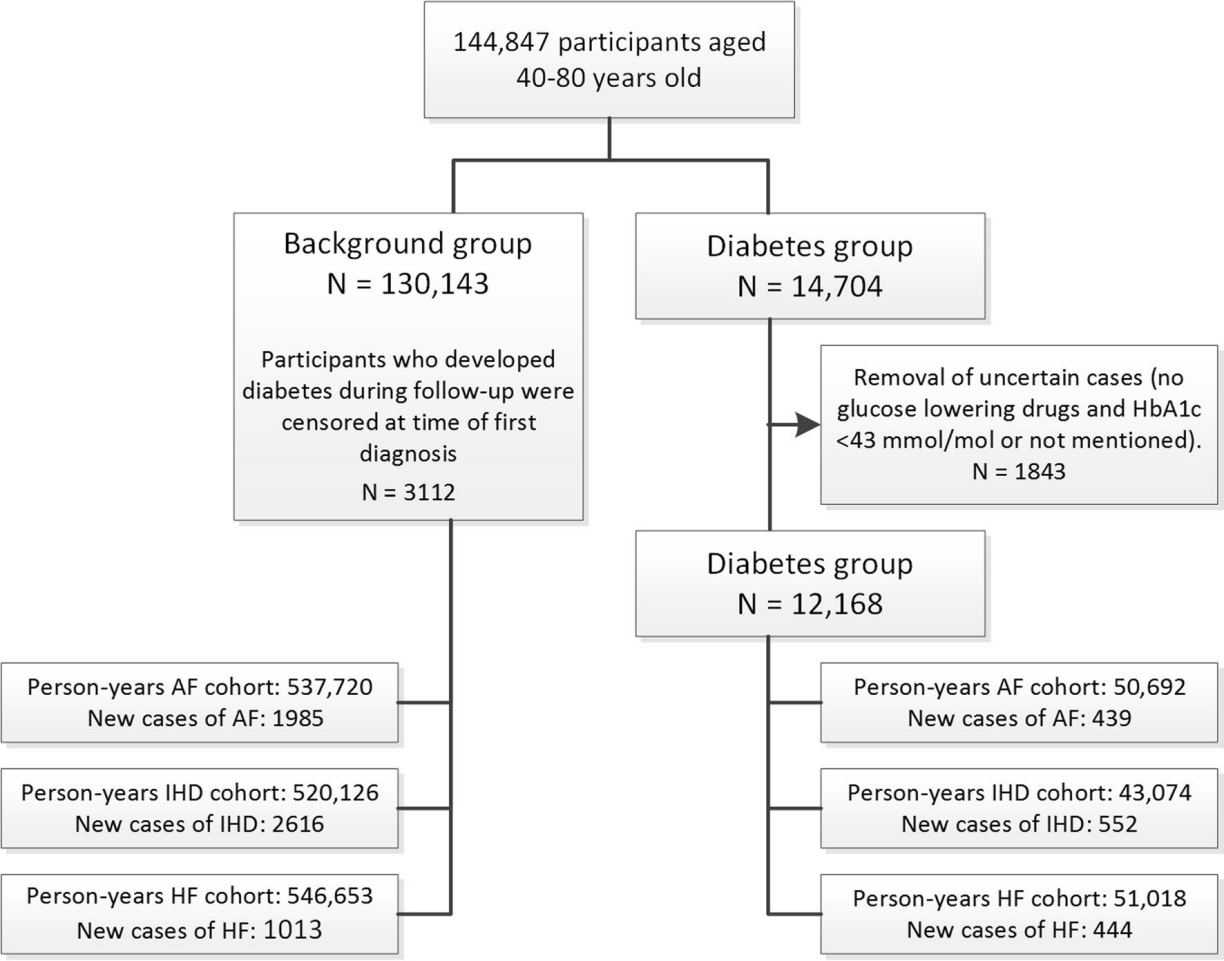
Flowchart of selection and composition of the cohort and sub-cohorts. AF: atrial fibrillation; IHD: ischaemic heart disease; HF: heart failure

New cases of atrial fibrillation, ischaemic heart disease and heart failure were identified by ICPC-coding (see Table 1). Because the aim was to estimate incidence of these three target diseases, patients who had prevalent atrial fibrillation, ischaemic heart disease and heart failure (in the GP electronic chart) either at baseline or developed it during follow-up were excluded from the cohort for that particular disease from that moment onwards, but remained in the cohort for the other outcomes (see also Fig. 2). For the calculation of event-free survival, patients with prevalent atrial fibrillation, ischaemic heart disease or heart failure were removed from the cohort and the three cardiovascular diseases were combined into a composite outcome.

**Table 1:** ICPC-codes used to identify outcomes

Atrial fibrillation		Ischaemic heart disease		Heart failure	
K78	Atrial fibrillation/atrial flutter	K74	Ischaemic heart disease with angina	K77	Heart failure
		K74.01	Unstable angina	K77.01	Acutely decompensated heart failure
		K74.02	Stable angina	K77.02	Chronic decompensated heart failure
		K75	Acute myocardial infarction		
		K76	Ischaemic heart disease without angina		
		K76.01	Coronary sclerosis		
		K76.02	Old myocardial infarction

### Data analysis

Baseline characteristics are presented as means (with standard deviations) or frequencies (with percentages). P-values were calculated using Pearson chi square tests for categorical variables and independent samples T-tests for continuous variables. Age- and sex-specific incidence of atrial fibrillation, ischaemic heart disease and heart failure are reported as cases per 1000 person-years according to diabetes status. Groups of 10-year age intervals were formed to estimate incidence by age. Incidence rate ratios were calculated with Poisson regression analysis, adjusting for age and sex, using the background group as controls and with person-time in years added as offset. Corresponding 95% confidence intervals were calculated using Byar’s approximation based on the Poisson distribution. After exclusion of patients with prevalent cardiovascular disease on baseline, Cox proportional hazards regression was used to investigate disease free survival in naïve individuals, adjusting for and sex and age at baseline, and for diabetes as time-varying variable. Atrial fibrillation, ischaemic heart disease and heart failure were applied as composite outcome, and the competing risk of death was taking into account. For the outcome cardiovascular disease, one would expect the hazard to change more as a function of age than as a function of time on study. Therefore, age was used as the time scale rather than follow-up time, so that individuals entered the analysis at their baseline age and exited at their event or censoring age. All analyses were conducted using IBM SPSS Statistics 25 and SAS Studio 3.8 (PROC PHREG).

## Results

During the study period, 144,847 participants were included in the cohort. Mean follow-up was 4.2 person-years. Diabetes diagnosis was considered uncertain in 1843 individuals. These were removed from the cohort, which left 130,143 (91%) individuals without diabetes and 12,168 (9%) with diabetes for analysis. Person-time and numbers of cases per sub-cohort are presented in Fig. 2 (participants with prevalent atrial fibrillation, ischaemic heart disease or heart failure at entry date were excluded from the analysis for that particular outcome only). Table 2 shows the baseline characteristics of patients with and without diabetes.

**Table 2:** The baseline descriptive statistics of the entire study population (aged 40–80 at start of cohort) contained in the Julius General Practice Network between 2014–2019

Variable	Diabetes	No diabetes	P-value
N	12,861	130,143	
Age in years (SD)	63.7 (10.3)	55.1 (10.7)	< 0.001
Female sex	47.3%	50.7%	< 0.001
*Comorbidities at baseline*			
Hypertension	7379 (57.4%)	25,484 (19.6%)	< 0.001
Atrial fibrillation	769 (6.0%)	2791 (2.1%)	< 0.001
Vascular disease			
Peripheral artery disease	923 (7.2%)	2627 (2.0%)	< 0.001
Any ischaemic heart disease/ angina pectoris	2479 (19.3%)	6601 (5.1%)	< 0.001
Prior myocardial infarction	1046 (8.1%)	2844 (2.2%)	< 0.001
Stroke	647 (5.0%)	2124 (1.6%)	< 0.001
TIA	462 (3.6%)	1829 (1.4%)	< 0.001
AAA	494 (3.8%)	1881 (1.4%)	< 0.001
Heart failure	767 (6.0%)	1273 (1.0%)	< 0.001
COPD	1286 (10.0%)	5342 (4.1%)	< 0.001
Impaired glucose tolerance	NA	1530 (1.2%)	
*Medication use at baseline*			
Oral glucose lowering drugs	9183 (71.4%)	NA	
Insulin	2611 (20.3%)	NA	
Antithrombotics, total	4073 (31.7%)	11,909 (9.2%)	< 0.001
Anticoagulants	928 (7.2%)	2785 (2.1%)	< 0.001
Vitamin K antagonists	911 (7.1%)	2660 (2.0%)	< 0.001
NOACs	21 (0.2%)	157 (0.1%)	0.191
Antiplatelet therapy	3255 (25.3%)	9022 (6.9%)	< 0.001
Aspirin	3112 (24.2%)	8621 (6.6%)	< 0.001
Clopidogrel	299 (2.3%)	812 (0.6%)	< 0.001
Prasugrel, ticagrelor	85 (0.7%)	292 (0.2%)	< 0.001
Diuretics	4337 (33.7%)	10,502 (8.1%)	< 0.001
Beta-blockers	4167 (32.4%)	12,758 (9.9%)	< 0.001
ACE-inhibitors/angiotensin-II-antagonists	6893 (53.6%)	14,515 (11.2%)	< 0.001
Calcium channel antagonists	2713 (21.1%)	6020 (4.6%)	< 0.001
Other antihypertensive drugs	186 (1.4%)	309 (0.2%)	< 0.001
Thyroid drugs			
Thyroid mimetics	680 (5.3%)	3373 (2.6%)	< 0.001
Thyroid inhibitors	20 (0.2%)	165 (0.1%)	0.387
Lipid lowering drugs/statins	8407 (65.4%)	12,813 (9.8%)	< 0.001
Antiarrhythmic drugs			
Class I	115 (0.9%)	541 (0.4%)	< 0.001
Class II	4167 (32.4%)	12,758 (9.9%)	< 0.001
Class III	52 (0.4%)	145 (0.1%)	< 0.001
Class IV	2713 (21.1%)	6020 (4.6%)	< 0.001

TIA: transient ischaemic attack; AAA: abdominal aortic aneurysm; COPD chronic obstructive pulmonary disease; NOAC: novel oral anticoagulant; ACE: angiotensin-converting enzyme; NA: not applicable

### Baseline characteristics of the study cohort

Patients with diabetes were generally older (63.7 years versus 55.1 years), more often male (52.7% versus 49.3%) and had more comorbidities at baseline compared to participants without diabetes. Of participants with diabetes, 71.4% used oral glucose lowering drugs, 20.3% used insulin.

### Incidence of atrial fibrillation

**Fig. 3:**
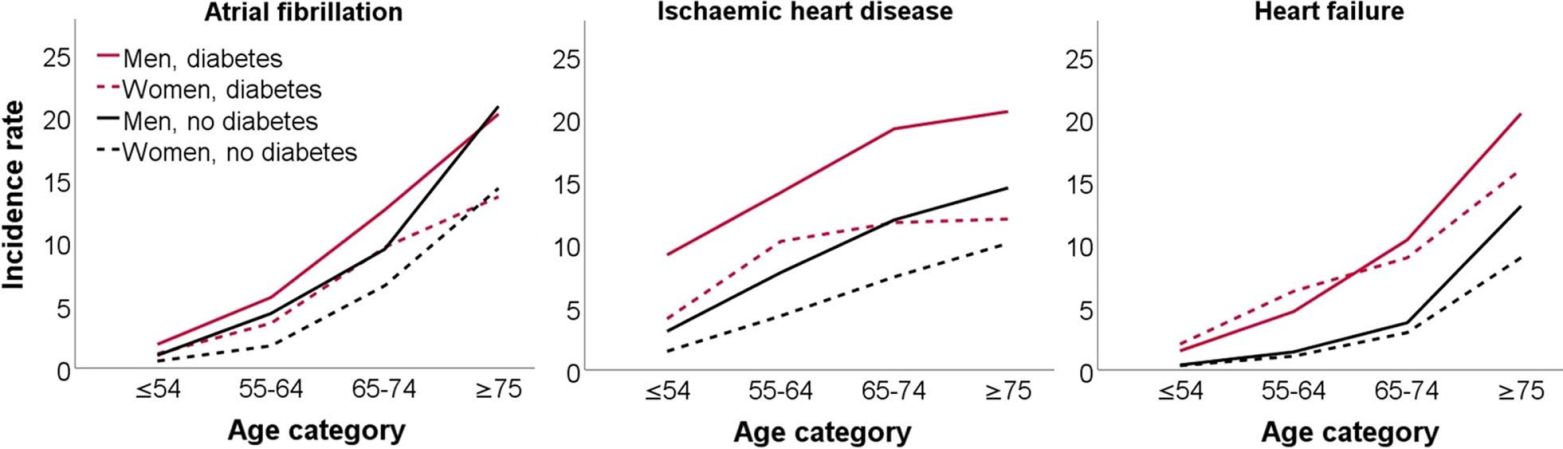
Incidence of cardiovascular diseases per 1000 person-years, for patients with and without diabetes, per age category

**Fig. 4:**
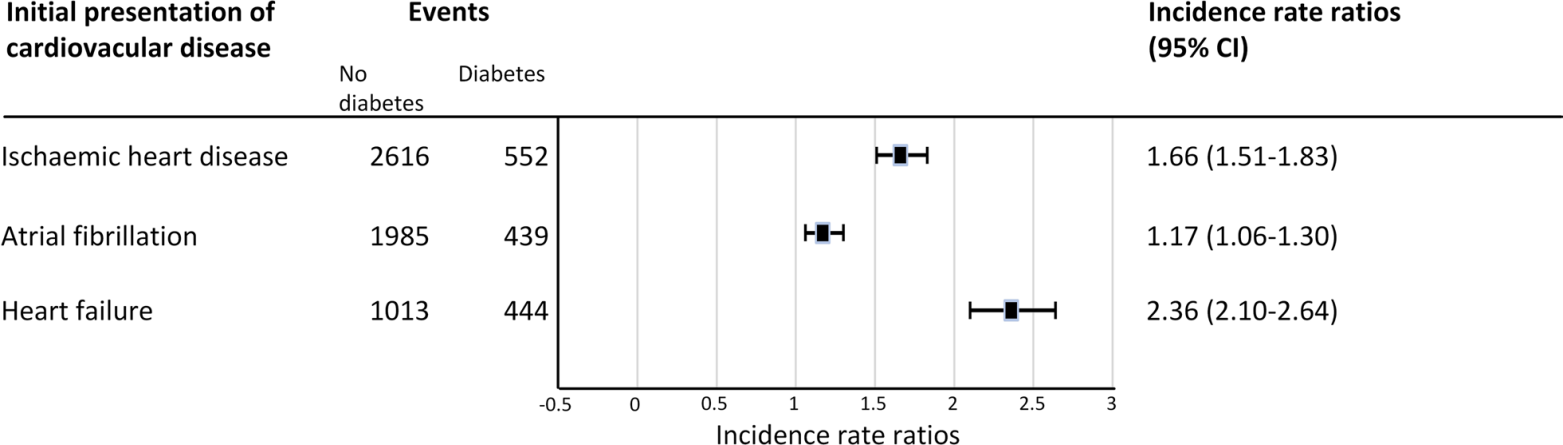
Age- and sex-adjusted incidence rate ratios for diabetics vs. non-diabetics of atrial fibrillation, ischaemic heart disease and heart failure

In men and women combined, incidence of atrial fibrillation is higher in subjects with diabetes compared to subjects without diabetes, although the incidences seem to converge in the highest age category. In participants without diabetes, incidence of atrial fibrillation is higher in men than in women at any given age. A similar difference between sexes is seen in the participants with diabetes, although the confidence intervals overlap in the youngest age categories, indicating that these differences are not statistically significant (see also Table 3 and Fig. 3) The overall incidence rate ratio of atrial fibrillation in diabetic patients vs. non-diabetic patients, adjusted for age and sex, was 1.17 (95% CI 1.06–1.30) (see Fig. 4).

**Table 3:** Incidence rate of atrial fibrillation per 1000 person-years

Men										
Age	Without diabetes				With diabetes					
	IR	95% CI	Cases	Person-time	IR	95% CI	Cases	Person-time	IRR	(95% CI)
40–54	1.00	0.84–1.18	135	134,774.42	1.88	0.95–3.35	10	5327.89	1.87	0.99- 3.56
55–64	4.34	3.87–4.85	301	69,410.39	5.61	4.08–7.54	41	7307.20	1.30	0.93–1.79
65–74	9.45	8.55–10.42	391	41,381.03	12.58	10.40–15.08	112	8904.65	1.33	1.08–1.64
≥ 75	20.84	18.66–23.20	324	15,549.86	20.21	16.50–24.52	98	4849.16	0.97	0.77–1.22

Women										
Age	Without diabetes				With diabetes					
	IR	95% CI	Cases	Person-time	IR	95% CI	Cases	Person-time	IRR	95% CI
40–54	0.54	0.43–0.68	72	132,156.27	1.13	0.41–2.50	5	4430.37	2.07	0.84–5.19
55–64	1.76	1.48–2.09	129	73,165.99	3.56	2.31–5.25	23	6466.80	2.02	1.30–3.14
65–74	6.55	5.86–7.29	325	49,643.81	9.60	7.64–11.92	78	8122.77	1.47	1.15–1.89
≥ 75	14.30	12.77–15.97	308	21,538.37	13.63	10.74–17.06	72	5283.53	0.95	0.74–1.23

Men and women										
Age	Without diabetes				With diabetes					
	IR	95% CI	Cases	Person-time	IR	95% CI	Cases	Person-time	IRR	95% CI
≤ 54	0.78	6.75–8.87	207	266,930.69	1.54	0.89–2.8	15	9758.27	1.98	1.17–3.35
55–64	3.01	2.74–3.31	430	142,676.38	4.65	3.61–5.90	64	13,773.99	1.54	1.19–2.01
65–74	7.87	7.31–8.46	716	91,024.84	11.16	9.67–12.83	190	17,027.42	1.42	1.21–1.67
≥ 75	17.04	15.75–18.41	632	37,088.23	16.78	14.39–19.45	170	10,132.69	0.98	0.83–1.17

CI: confidence interval; IR: incidence rate; IRR: incidence rate ratio

Incidence rate per 1000 person-years. Person-time in years

### Incidence of ischaemic heart disease

Figure 3 and Table 4 show that differences in risk between patients with and without diabetes are most pronounced in the lower age categories (Table 4 and Fig. 3). In men and women with diabetes, the incidence rate reaches its peak in the 65–74 age category and remains largely stable afterwards (as evidenced by the flattening curves). In subjects without diabetes, incidence rate of ischaemic heart disease continues to increase gradually with increasing age. In both subject with and without diabetes, incidence of ischaemic heart disease is significantly higher in men than in women at any given age (except in patients with diabetes aged 55–64 years old). The overall incidence rate ratio for ischaemic heart disease in patients with vs. without diabetes, adjusted for age and sex, is 1.66 (95% CI 1.51–1.83) (Fig. 4).

**Table 4:** Incidence rate of ischaemic heart disease per 1000 person-years

Men										
Age	Without diabetes				With diabetes					
	IR	95% CI	Cases	Person-time	IR	95% CI	Cases	Person-time	IRR	95% CI
≤ 54	3.06	2.77–3.37	406	132,643.08	9.14	6.64–12.28	44	4812.71	2.99	2.19–4.08
55–64	7.74	7.08–8.44	510	65,918.29	14.11	11.27–17.45	85	6024.63	1.82	1.45–2.29
65–74	11.93	10.85–13.09	446	37,381.03	19.18	16.07–22.72	134	6984.80	1.61	1.33–1.95
≥ 75	14.48	12.52–16.65	196	13,538.92	20.55	16.19–25.72	76	3698.83	1.42	1.09–1.85

Women										
Age	Without diabetes				With diabetes					
	IR	95% CI	Cases	Person-time	IR	95% CI	Cases	Person-time	IRR	95% CI
≤ 54	1.47	1.27–1.69	193	131,169.68	4.05	2.36–6.49	17	4194.19	2.76	1.69–4.52
55–64	4.28	3.82–4.79	305	71,182.35	10.22	7.78–13.19	59	5771.95	2.39	1.81–3.15
65–74	7.40	6.65–8.21	353	47,722.13	11.72	9.33–14.52	83	7084.93	1.58	1.25–2.01
≥ 75	10.06	8.74–11.53	207	20,571.43	11.99	9.01–15.65	54	4502.30	1.19	0.88–1.61

Both										
Age	Without diabetes				With diabetes					
	IR	95% CI	Cases	Person-time	IR	95% CI	Cases	Person-time	IRR	95% CI
≤ 54	2.27	2.09–2.46	599	263,812.76	6.78	5.18–8.70	61	9006.90	2.98	2.29–3.88
55–64	5.95	5.54–6.37	815	137,100.64	12.21	10.29–14.37	144	11,796.57	2.05	1.72–2.45
65–74	9.39	8.75–10.06	799	85,103.16	15.43	13.44–17.62	217	14,067.73	1.64	1.41–1.91
≥ 75	11.81	10.96–13.03	403	34,110.35	15.85	13.24–18.82	130	8201.13	1.34	1.10–1.64

CI: confidence interval; IR: incidence rate; IRR: incidence rate ratio

Incidence rate per 1000 person-years. Person-time in years

### Incidence of heart failure

The incidence of heart failure is significantly higher in individuals with diabetes across all age categories, for both men and women (see Table 5 and Fig. 3). The incidence rate ratio for heart failure in patients with diabetes vs. patients without diabetes, adjusted for age and sex, is 2.36 (95% CI 2.10–2.64) (Fig. 4). Incidence of heart failure reaches its peak in the oldest age category and is fairly similar for men and women.

**Table 5:** Incidence rate of heart failure per 1000 person-years

Men										
Age	Without diabetes				With diabetes					
	IR	95% CI	Cases	Person-time	IR	95% CI	Cases	Person-time	IRR	95% CI
≤ 54	0.38	0.27–0.50	52	135,654.03	1.50	0.65–2.96	8	5322.10	3.92	1.86–8.25
55–64	1.42	1.16–1.73	101	70,976.84	4.63	3.21–6.47	34	7343.78	3.25	2.21–4.80
65–74	3.75	3.19–4.37	163	43,530.38	10.34	8.37–12.64	95	9185.63	2.76	2.15–3.56
≥ 75	13.03	11.37–14.87	220	16,880.96	20.40	16.62–24.79	101	4951.17	1.57	1.28–1.98

Women										
Age	Without diabetes				With diabetes					
	IR	95% CI	Cases	Person-time	IR	95% CI	Cases	Person-time	IRR	95% CI
≤ 54	0.33	0.24–0.45	44	132,338.64	2.04	0.93–3.87	9	4412.07	6.14	3.00–12.57
55–64	1.09	0.86–1.35	80	73,697.06	6.23	4.46–8.51	40	6400.99	5.76	3.94–8.41
65–74	2.96	2.51–3.48	151	50,947.58	8.92	6.98–11.23	72	8072.97	3.01	2.27–3.99
≥ 75	8.93	7.74–10.25	202	22,628.39	15.59	12.74–19.72	85	5329.83	1.79	1.39–2.30

Both										
Age	Without diabetes				With diabetes					
	IR	95% CI	Cases	Person-time	IR	95% CI	Cases	Person-time	IRR	95% CI
≤ 54	0.36	0.29–0.44	96	267,992.67	1.75	1.02–2.80	17	9734.17	4.88	2.91–8.17
55–64	1.25	1.08–1.45	181	144,673.90	5.38	4.23–6.76	74	13,744.77	4.30	3.28–5.64
65–74	3.32	2.97–3.71	314	94,477.96	9.68	8.26–11.26	167	17,258.61	2.91	2.41–3.51
≥ 75	10.68	9.69–11.75	422	39,509.35	18.09	15.58–20.89	186	10,281.00	1.69	1.43–2.01

CI: confidence interval; IR: incidence rate; IRR: incidence rate ratio

Incidence rate per 1000 person-years. Person-time in years

Overall, the highest incidence rate ratio was observed for heart failure (2.36), followed by ischaemic heart disease (1.66) and atrial fibrillation (1.17) (Fig. 4).

### Time between events and disease-free survival

**Fig. 5:**
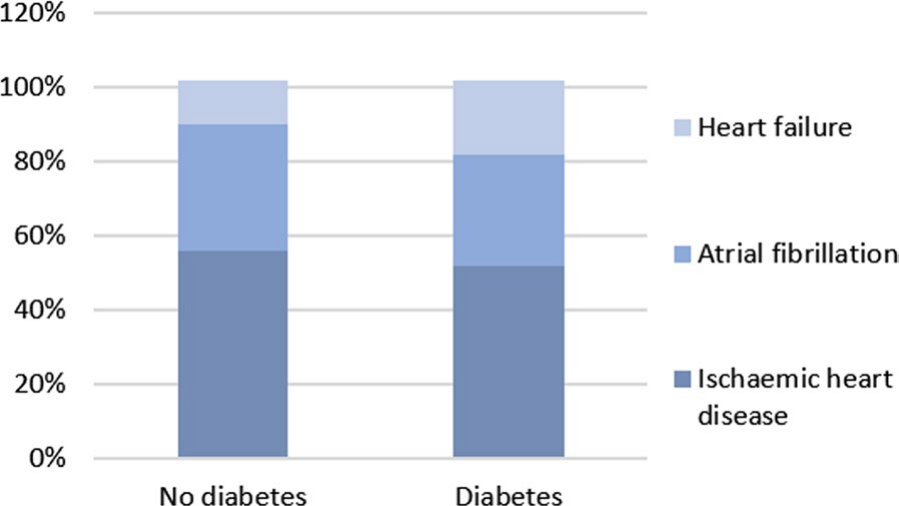
Distribution of the initial manifestation of cardiovascular disease in individuals with and without diabetes. Total percentage exceeds 100% because some individuals were diagnosed with more than one cardiovascular disease at initial presentation (i.e. atrial fibrillation and heart failure)

After exclusion of patients with any cardiovascular disease at baseline, 5420 participants developed at least one cardiovascular disease during the average 4.1 years of follow-up. The mean follow-up after the initial manifestation of CVD was another 2.2 years, during which 476 individuals developed a second CVD and 27 developed a third CVD (Fig. 5 and Additional file 1: Table S1).

Ischaemic heart disease was the most common first presentation in patients with and without diabetes. However, of the 940 patients with type 2 diabetes who had cardiovascular events, heart failure was the first presentation in 191 (20%) patients (compared to 13% in patients without diabetes). Time between first and second CVD was relatively short; 6–12 months (Additional file 1: Table S2).

**Fig. 6:**
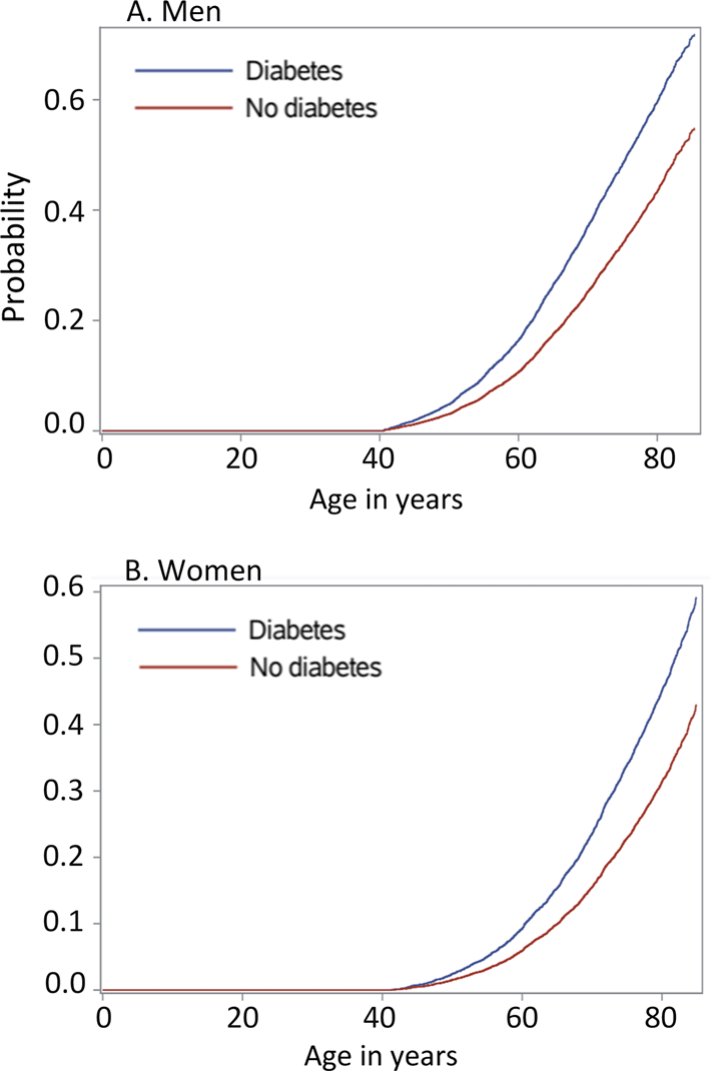
Proportion of patients with at least one cardiovascular event up to 80 years, for patients with and without diabetes

Figure 6 A and B show the cumulative incidence curve for men and women with or without diabetes, estimating the proportion of patients with events up to 80 years for patients with and without diabetes at age 40.

## Discussion

Our results, based on primary care data of 130,143 individuals without diabetes and 12,168 diabetes patients followed-up for a mean of 4.2 years, reaffirm that the incidence of atrial fibrillation, ischaemic heart disease and heart failure is higher in individuals with diabetes than in individuals without diabetes. The highest incidence rate ratio was observed for heart failure (2.36), followed by ischaemic heart disease (1.66) and atrial fibrillation (1.17).

### Comparison to other studies

Several other cohorts have investigated incidence of cardiovascular disease in patients with diabetes. However, now that newer glucose lowering drugs are used more frequently, our data provide much needed up-to-date estimates of cardiovascular disease incidence in the general population. In addition, earlier studies have often excluded individuals who had experienced any type of cardiovascular disease before the outcome of interest and are therefore expected to find lower estimates, especially since heart failure is strongly associated with age and pre-existing cardiovascular disease [1].

In a Danish cohort study including over 5 million inhabitants, the incidence rate ratio of atrial fibrillation adjusted for calendar year, age, hypertension, chronic obstructive pulmonary disease, ischaemic heart disease, chronic heart failure, chronic kidney disease, valvular heart disease and hyperthyroidism was 1.17 (95% CI 1.16–1.19) for both men and women with diabetes, compared to a background population without diabetes [17]. As is the case in the present study, the risk was more pronounced in the younger diabetes patients, who had a more than twofold increase in risk. Similarly, in 2018, a meta-analysis including 31 cohort studies showed an overall increased risk of 1.28 (95% CI 1.22–1.35) for developing atrial fibrillation with diabetes, although the included studies were conflicting and there was substantial heterogeneity. These discrepancies may be attributed to differences in study populations, as the studies included in the meta-analysis were often smaller and, in some cases, performed in selected cohorts.

Since the Framingham Heart Study first demonstrated this in 1978, other cohorts have consistently shown that diabetes confers an increased risk for ischaemic heart disease as well as cardiac mortality, although incidences differ based on population studied and validation methods used. Incidence is ideally estimated in long term community-based cohorts (or representative samples of such a cohort) defined by a certain geographical area. One such cohort is the population-based Atherosclerosis Risk in Communities (ARIC) study, in which patients without prior MI were followed up for an average of 9 years (125,998 person-years). The event rate of fatal coronary heart disease or non-fatal MI was 10.8/1000 person-years in patients with diabetes compared to 3.9/1000 person-years in patients without diabetes [21]. Our cohort showed slightly higher incidence rates, as our definition of ischaemic heart disease was broader, including angina pectoris as well. A very large, recent cohort with 1.9 million individuals, which included linked data from primary care, hospital admissions, disease registries, and death certificate records, found that type 2 diabetes was associated with higher incidence of stable angina (1.62 [95% CI 1.49–1.77]) and non-fatal myocardial infarction (1.54 [1.42–1.67]) [1].

Diabetes and heart failure are strongly connected, as has been reported by several others: diabetes is associated with higher rates of heart failure, [22] while heart failure is also associated with a higher incidence of new onset diabetes [23]. Risk factors for heart failure (older age, obesity, hypertension, chronic kidney disease, sleep apnoea, dyslipidaemia) also cluster in patients with diabetes, and heart failure is remarkably often the first presentation of CV disease in this population [1]. Interestingly, glucometabolic abnormalities and obesity portend a stronger risk factor for heart failure with a preserved ejection fraction (HFpEF) than for heart failure with a reduced ejection fraction (HFrEF) [24]. Biomarkers of myocardial stretch, inflammation or injury confer comparable predictive value in subjects with and without diabetes [25]. In the Framingham Heart Study, frequency of congestive heart failure was doubled in diabetic men (aged 45 to 74 years) compared to the nondiabetic cohort, and diabetic women had a fivefold increased risk [26]. Results of later studies have been variable with regards to the difference in relative risk between the sexes. A recent meta-analysis of 47 cohort studies, including 12 million individuals, found that relative risks for heart failure associated with type 2 diabetes were 1.95 (1.70, 2.22) in women and 1.74 (1.55, 1.95) in men, with a pooled men- to-women ratio of 1.09 (1.05, 1.13), indicating a significant difference [27]. In the current cohort, incidence rate ratios adjusted for age were 2.13 (95% CI 1.82–2.49) in diabetic vs. non-diabetic men and 2.65 (95% CI 2.24–3.13) in diabetic vs. non-diabetic women, with overlapping confidence intervals. Our finding that younger diabetes patients are also at risk of heart failure was reported previously in the U.K. Prospective Diabetes Study (UKPDS) including 4,585 patients with newly diagnosed diabetes younger than 65 years at diagnosis found heart failure incidence rates of 2.3–11.9 per 1000 patient-years during 10 years of follow-up [28].

Our data reiterate that diabetes confers very strong—if not the strongest—risk for new onset heart failure, in comparison to other diseases in the CV spectrum.

### Cardiovascular disease by age and sex

Although the absolute incidence rates of all three cardiovascular diseases in this study increased steadily with age for both diabetics and non-diabetics, the incidence rate ratios declined with ageing, with the highest relative rates in the youngest diabetes patients. A stronger association between diabetes and cardiovascular diseases in younger patients was also noted in other studies, including the meta-analysis of cohort studies in the Emerging Risk Factors Collaboration [1, 18]. This demonstrates that diabetes may not only increase the risk of cardiovascular disease, but also accelerate its development and progression. A growing disease burden is to be expected, as the years lived with symptoms increase while the young diabetes population ages.

In patients without diabetes, the incidence of CV diseases was consistently higher in men than in women, as reported previously. In diabetics, this sex difference was present but not significant, except for ischaemic heart disease. In fact, ratios of CVD incidence rates in diabetics vs. non-diabetics are as high in women as they are in men, suggesting that diabetes negates some of the cardiovascular protection that (premenopausal) women normally have.

## Strengths and limitations

The well-organized nature of health care in the Netherlands, with GPs in gatekeepers’ positions, makes routine primary care data well suited for estimation of incidence of cardiovascular diseases. As patients increasingly receive care in an primary care setting and may not visit a hospital for cardiovascular diseases unless an acute event occurs, primary care data is far more inclusive than hospital care data, making our data generalizable to the overall population. Furthermore, because consecutive hospitalizations of the same individual for the same disease are often counted as separate events in hospital registries, hospital data are unsuited to inform on incidence.

Some limitations should be noted. Firstly, we relied on ICPC-coding for the evaluation of presence or absence of outcome, without applying further case validation. However, previous studies have shown that the database produces reliable quantitative estimates of symptom incidence, disease prevalence, referral and prescription rates [20]. Nevertheless, a risk of misclassification exists and the numbers presented here are likely to be an underestimation of the true incidence and prevalence of cardiovascular diseases in patients with diabetes. Opportunistic screening studies have shown that a full diagnostic work-up (including symptom questionnaires, natriuretic peptide measurement and echocardiography) can reveal previously unrecognized heart failure in up to 27% of diabetes patients (85% HFpEF and 15% HFrEF, cut-off LVEF45%) [29]. Previously unrecognized ischaemic heart disease is even more prevalent, found in up to 60% of diabetes patients screened with coronary CT-scanning [30]. In the prospective DIAD study, 522 diabetes patients were assigned to adenosine stress myocardial perfusion imaging and silent ischaemia was found in 22 percent of patients, with large defects identified in 6 percent [31]. Of the general population, about 7% has silent ischaemia [32]. Atrial fibrillation may similarly remain undiagnosed, especially when it has a paroxysmal character or when it is asymptomatic, commonly in patients with new-onset atrial fibrillation or in patients using beta-blockers. The exact incidence of ‘silent’ atrial fibrillation is unknown, but the Belgrade Atrial Fibrillation study showed that a history of diabetes is an independent predictor of asymptomatic presentation of first-diagnosed non-valvular AF [33]. Another possible reason for underdiagnosis of atrial fibrillation, ischaemic heart disease and heart failure in diabetes patients is the similarity between symptoms suggestive of cardiovascular disease and those that commonly accompany diabetes (e.g. exercise intolerance, fatigue, dyspnea on exertion).

Secondly, even though the cohort was large, numbers of events in lower age categories were relatively small particularly in the diabetes group, resulting in rather wide confidence intervals.

Also, we did not adjust for use of medication, even though clear baseline differences were noted. Patients with diabetes are typically treated with statins and anti-hypertensive medication, which will have a beneficial effect on the risk of developing cardiovascular diseases. As such, our data can only be interpreted as a reflection of the incidence of CVD in real world patients, rather than as an argument for diabetes as an independent risk factor.

Lastly, although type 1 and type 2 diabetes are different pathological entities, they were not analyzed separately. This is because of coding issues; the ICPC code for diabetes (T90) can be used for both types and further classification into type 1 (T90.01) or type 2 (T90.02) is optional and not routinely or accurately done, so that some patients are classified as neither of both subtypes. Including both types of diabetes minimizes the risk of misclassification of exposure status but may have influenced the incidence estimates. Based on previous studies cardiovascular disease in type 1 diabetes differs from type 2 diabetes mellitus, in that it presents at a younger age and affects men and women equally [34]. However, of adults with diagnosed diabetes, type 1 accounts for around 5% of the cases, and type 2 for 95% [35] so the influence of these differences on the estimations presented here is likely limited.

## Conclusion

There is a clear association between diabetes and the incidence of major chronic CV disease, notably heart failure has a more than twice increased incidence rate. Patients with diabetes develop cardiovascular diseases not only more often, but also at a younger age. Our data may prompt physicians caring for patients with diabetes to pro-actively screen for CVD, and heart failure in particular.

## Supplementary Information

**Additional file 1: Additional Tables.**

## Data Availability

Following Dutch privacy regulations and JGPN terms and conditions, our data will be returned to the JGPN or destructed. Requests for data sharing can be made from the JGPN committee, which will make data available under strict conditions.
