# Supplementary material for: Incidence of atrial fibrillation, ischaemic heart disease and heart failure in patients with diabetes
Source: Cardiovasc Diabetol. 2021 Jun 16;20:123. doi: 10.1186/s12933-021-01313-7 (PMC8210360; doi:10.1186/s12933-021-01313-7)
Supplement: Supplementary file 1 — Additional file 1: Additional Tables. [file 12933_2021_1313_MOESM1_ESM.docx]

**Appendix**

| **Table 1. Number of new cardiovascular diseases and initial manifestations in individuals with diabetes who were free from cardiovascular disease at baseline** | | | | | | |
| --- | --- | --- | --- | --- | --- | --- |
|  | **Total** | | **No diabetes** | | **Diabetes** | |
| **Number of new CVDs** | **N** | **%** | **N** | **%** | **N** | **%** |
| Any number | 5420 |  | 4480 |  | 940 |  |
| 1 CVD | 4917 | 91% | 4108 | 92% | 809 | 86% |
| 2 CVDs | 476 | 9% | 352 | 8% | 124 | 13% |
| 3 CVDs | 27 | 0.5% | 20 | 0.4% | 7 | 0.7% |
|  |  |  |  |  |  |  |
| **First CVD** | **N** | **%*** | **N** | **%*** | **N** | **%*** |
| Ischaemic heart disease | 2978 | 55% | 2490 | 56% | 488 | 52% |
| Atrial fibrillation | 1792 | 33% | 1507 | 34% | 285 | 30% |
| Heart failure | 749 | 14% | 558 | 12% | 191 | 20% |
| CVD cardiovascular disease.  *Total percentage exceeds 100% because some individuals were diagnosed with more than 1 CVD at initial presentation (i.e. atrial fibrillation and heart failure). | | | | | | |

| **Table 2. Average time to development of a second cardiovascular disease, during mean follow-up of 2.2 years after initial manifestation** | | | | | | | | | | | | |
| --- | --- | --- | --- | --- | --- | --- | --- | --- | --- | --- | --- | --- |
|  | | **No diabetes** | | | | | **Diabetes** | | | | | |
| **Initial manifestation** | |  | **Second CVD** | | **Time to second CVD** | |  | | **Second CVD** | | **Time to second CVD** | |
|  | |  | **N** | **%** |  |  |  |  | **N** | **%** |  |  |
| **Ischaemic heart disease (N=2490)** | | | | | | | **Ischaemic heart disease (N=488)** | | | | | |
|  | Atrial fibrillation | | 91 | 3.7% | 12 months | | Atrial fibrillation | | 13 | 2.6% | 8 months | |
|  | Heart failure | | 58 | 2.3% | 8 months | | Heart failure | | 37 | 7.6% | 9 months | |
| **Atrial fibrillation (N=1507)** | | | | |  |  | **Atrial fibrillation (N=285)** | | | | | |
|  | Heart failure | | 89 | 5.9% | 11 months | | Heart failure | | 41 | 14.3% | 11 months | |
| **Heart failure (N=558)** | | | | |  |  | **Heart failure (N=191)** | | | | | |
|  | Atrial fibrillation | | 49 | 8.8% | 6 months | | Atrial fibrillation | | 13 | 6.8% | 9 months | |
| Time from heart failure and atrial fibrillation to ischaemic heart disease is not shown here. Atrial fibrillation and heart failure are more often ‘end stage’ diseases and patients who develop these conditions are usually screened for ischaemic heart disease at the time of diagnosis, so that diagnoses are set simultaneously and follow-up time is very short. | | | | | | | | | | | | |
